# Research progress on the mechanism of transcutaneous electrical acupoint stimulation in the perioperative period

**DOI:** 10.3389/fneur.2025.1563681

**Published:** 2025-04-11

**Authors:** Xiaobo Jiang, Mengqi Li, Yuanjian Tang, Jing Hu, Xiangmu Gai, Chenfeng Zhang, Tie Li, Yanxin Wang, Hongfeng Wang

**Affiliations:** ^1^College of Acupuncture and Tuina, Changchun University of Chinese Medicine, Changchun, China; ^2^College of Traditional Chinese Medicine, Changchun University of Chinese Medicine, Changchun, China; ^3^Department of Paediatric Endocrinology, The First Hospital of Jilin University, Changchun, China; ^4^Department of Cardiovascular Rehabilitation, The Third Clinical Affiliated Hospital of Changchun University of Chinese Medicine, Changchun, China; ^5^Northeast Asia Institute of Traditional Chinese Medicine, Changchun University of Chinese Medicine, Changchun, China

**Keywords:** transcutaneous electrical acupoint stimulation, perioperative period, mechanism, surgery, rehabilitation

## Abstract

Transcutaneous Electrical Acupoint Stimulation (TEAS) is a non-invasive therapeutic approach that combines modern electrophysiological technology with traditional Chinese medicine principles. Over the past few decades, TEAS has become a significant medical intervention during the perioperative period and its mechanisms have seen a continuous development. These mechanisms span across multiple aspects, including the regulation of the neuroendocrine network system, inhibition of oxidative stress, alleviation of immune suppression, regulation of endothelial function, and inhibition of coagulation and fibrinolysis system activation. This article aims to summarize and elaborate the mechanisms of TEAS in the perioperative period and to discuss current research challenges. Future studies should strive to optimize treatment protocols to promote the research on the mechanisms of TEAS in the perioperative period, subsequently offering patients safer and more effective treatment alternatives.

## 1 Introduction

The perioperative period refers to the duration from decision to proceed with surgery to the completion of all related treatments, including preoperative, intraoperative, and postoperative phases (1). TEAS is a therapeutic approach that combines modern electrophysiological technologies with the principles traditional acupuncture. Moreover, the theoretical foundation of TEAS is rooted in the traditional Chinese medical concepts of meridians and acupuncture points, as further strengthened in the ancient medical text, Huangdi Neijing, in terms of its significance in sustaining physiological equilibrium. Currently, TEAS is widely used in the perioperative period, demonstrating considerable therapeutic efficacy. Specifically, a large amount of research have shown the application of TEAS in alleviating various perioperative symptoms, such as surgical pain, postoperative cognitive dysfunction (POCD), gastrointestinal issues, and sleep disturbances (2, 3), mainly used for preoperative rehabilitation, intraoperative anesthesia cooperation and postoperative complications treatment.

With the increasing understanding of its clinical efficacy, researchers have begun to focus more on the mechanisms of TEAS during the perioperative period. Studies have indicated that TEAS could inhibit inflammatory responses, modulate the release of endogenous analgesic substances, and modify the stress responses during the perioperative period (4–6). Nevertheless, a comprehensive review on this topic is still marginal. Therefore, to fill this research gap, this article aims to summarize and specify the mechanisms of TEAS in the perioperative period, so as to provide a theoretical basis and treatment strategies for promoting the clinical application of TEAS during the perioperative period.

## 2 Regulation of the neuroendocrine network system

### 2.1 Regulation of the opioid system and pain-inducing substance secretion

Opiorphin, a natural opioid peptide found in the spinal cord, serves as a potent analgesic. In a randomized single-blind clinical trial, during the perioperative period, the application of TEAS could promotes the expression of opiorphin. Through the opioid system, TEAS inhibits the degradation of encephalin by neutral endopeptidase and aminopeptidase N in patients undergoing general anesthesia for colorectal surgery, thereby enhancing the analgesic effect of encephalin and achieving a sedative clinical outcome (4). What’s more, β-Endorphin (β-EP), widely distributed in the central nervous system, acts as an opioid receptor agonist and plays a crucial role in pain regulation. Its increase will enhance the body’s analgesic response. During the investigation on the analgesic effect of TEAS during labor, it was observed that TEAS could elevate the plasma β-EP levels and enhance analgesic effect (7).

Serotonin (5-hydroxytryptamine, 5-HT) and prostaglandin E2 are predominantly secreted by injured tissues and may directly act on nociceptive sensory neurons, significantly impacting pain generation and conduction of pain. Furthermore, the application of TEAS at a frequency of 2/100 Hz as an adjunct to anesthesia could inhibit the secretion of 5-HT and prostaglandin E2 by various cells in patients undergoing thoracic surgery. This reduces the transmission of noxious stimuli to the central nervous system, activates analgesic mechanisms, and facilitates the release of opioid peptides such as β-EP, thereby alleviating pain in the patients (5).

Moreover, 5-HT plays a role in the regulation of gastrointestinal motility and several endocrine functions. Emetic molecules such as 5-HT which will increase under trauma or anesthesia, could directly stimulate the vomiting center of medulla oblongata, or activate the peripheral receptors in the gastrointestinal mucosa (8). A randomized controlled clinical trial proved that TEAS could inhibit the release of 5-HT in the central nervous system of patients undergoing gastric cancer surgery, resulting in improved POGD and reduced postoperative pain (8). Furthermore, TEAS might decrease the levels of vasoactive intestinal peptide, somatostatin, and neurotensin in patients undergoing gastrointestinal surgery, while simultaneously increasing the levels of motilin, gastrin, cholecystokinin, and ghrelin. These effects facilitate the recovery of gastrointestinal function and indirectly alleviate discomfort in patients after gastric cancer surgery (9, 10).

### 2.2 Regulation of neurometabolism

Increased levels of serum S-100β and neuron-specific enolase, along with a decrease in brain-derived neurotrophic factor, serve as indicators of neural cell injury and are closely associated with POCD (11). TEAS treatment at a frequency of 2/100 Hz could inhibit the levels of neuron-specific enolase and S100β protein in the serum of elderly patients undergoing total knee arthroplasty. What’s more, the level of brain-derived neurotrophic factor may be enhanced, improving neurometabolic function and having a positive effect on the prevention of POCD (12).

Tau protein, a microtubule-associated protein, is essential for maintaining the stability of microtubules within axons. Generally, after a single-port thoracoscopic lobectomy in elderly patients, the level of tau protein (p-tau) is abnormally raised. Consequently, the formation of neurofibrillary tangles within the brain parenchyma is accelerated, which is closely related to the formation of cognitive impairment. Accordingly, researchers have identified that TEAS is able to reduce serum p-tau levels in elderly patients, thereby improving POCD (13).

Melatonin is a facilitator of the memory process, and its reduction is associated with damage to the olfactory epithelial nerves (14). During the perioperative period, the use of sevoflurane anesthesia could lead to short-term olfactory recognition impairment, memory decline, and a decrease in melatonin levels (15). The application of TEAS in patients under sevoflurane anesthesia has been investigated to increase melatonin levels and aid in the recovery of olfactory memory. Furthermore, plasma melatonin plays a role in guiding sleep. A study on TEAS indicated that stimulation at Shenmen (HT7), Sanyinjiao (SP6), and Neiguan (PC6) can increase perioperative plasma melatonin concentrations among 30 living kidney donors, thereby reducing the incidence of postoperative sleep disturbances (16).

### 2.3 Enhancement of autonomic nervous system regulation

Autonomic imbalance during the perioperative period may lead to POGD, characterized by enhanced sympathetic excitability and reduced parasympathetic or vagal nerve activity postoperatively (17). The sympathetic nervous system inhibits gastric motility and emptying by releasing adrenaline, whereas the vagus nerve promotes gastric motility and emptying through the release of epinephrine (EPI) (18). Consequently, a decrease in digestive function may result from reduced parasympathetic nerve function weakening gastrointestinal peristalsis and increased sympathetic nerve excitability inhibiting gastrointestinal motility (17). Furthermore, heart rate variability analysis primarily reflects autonomic nervous regulation. Low-frequency power (LF) reflects sympathetic nervous activity, while high-frequency power (HF) reflects parasympathetic or vagal nerve activity. The LF/HF ratio indicates the balance between these two systems. The root mean square of successive R-R interval differences reflects parasympathetic nervous tension, while the standard deviation of 24-h R-R intervals reflects the combined effects of parasympathetic and sympathetic nervous activity (19). To sum up, through reducing LF/HF, increasing the patient’s 24-h R-R interval standard deviation, HF, and the root mean square of the successive R-R interval difference, TEAS could be utilized to reduce sympathetic nerve excitability in the early postoperative phase, restore parasympathetic nerve function, and ultimately prevent POGD (17, 20).

### 2.4 Inhibition of inflammatory mediators

Early inflammatory responses during the perioperative period are risk factors for postoperative pulmonary complications, with patients undergoing surgeries closer to the chest and abdomen being more likely to develop such complications (6, 21). What’s more, inflammatory mediators such as tumor necrosis factor-alpha (TNF-α), interleukin-1 (IL-1), and C-reactive protein (CRP) are positively correlated with the degree of inflammation. Accordingly, TEAS could be adopted to reduce the level of inflammatory factors, thereby decreasing the severity of inflammation. In particular, the application of TEAS at Gegu (LI4), Neiguan (PC6), Houxi (SI3), and Zhigou (SJ6) could reduce IL-6 and IL-10 levels in patients undergoing thoracoscopic lung lobectomy, alleviating inflammatory responses caused by single-lung ventilation and preventing pulmonary complications (6). Similarly, TEAS at a frequency of 2/100 Hz may lower TNF-α, IL-6, and CRP levels in esophageal cancer patients, reducing the risk of postoperative pulmonary complications and inflammation (21). Additionally, laparoscopic colorectal surgery is frequently influenced by the stimulation of the inflammatory response, potentially resulting in postoperative complications, including impaired gastrointestinal function and intestinal paralysis (22). In a randomized, double-blind clinical trial, involving 30 colorectal cancer patients undergoing radical surgery, researchers applied TEAS at a frequency of 2 Hz. This treatment reduced the patients’ levels of TNF-α, IL-1, and IL-6, thereby mitigating the inflammatory response (23).

## 3 Inhibition of oxidative stress

In perioperative patients, stress reactions may result in the production of excessive reactive oxygen species, leading to cellular dysfunction and ultimately causing irreversible cell damage and death (24). During the process, patients may exhibit clinical manifestations such as blood pressure fluctuations and sleep disturbances, which may negatively affect postoperative recovery (25, 26). Additionally, the human body could generate various stress factors, including norepinephrine, EPI, adrenocorticotropic hormone, angiotensin II, cortisol (Cor), and CRP (27). The application of TEAS in patients undergoing laparoscopic cholecystectomy has been shown to significantly reduce the levels of NE, EPI, angiotensin II, CRP, and Cor, thereby alleviating the stress response (28, 29). Furthermore, the application of TEAS prior to anesthesia induction in hypertensive patients may mitigate the increase in adrenocorticotropic hormone and Cor, thereby aiding in the maintenance of circulatory stability and diminishing the stress response (25).

Secretory immunoglobulin A (SIgA) is an important salivary immune molecule that plays a key role in mucosal immunity. Trauma induces a stress response in the body, which alters mucosal immune function and increases SIgA concentration. Salivary amylase (sAA) is a specific indicator of stress. The level of sAA is directly proportional to the intensity of pain and the degree of discomfort caused by pain. Postoperative patients often experience varying degrees of tension and stress. During general anesthesia for colorectal surgery, the application of TEAS has been shown to reduce levels of SIgA, sAA, and Cor, effectively alleviating the stress response (4).

In the early postoperative period, patients frequently undergo sleep disturbances, which lead to an increase in reactive oxygen species and subsequently trigger oxidative stress. This process results in a decreased expression of nuclear factor erythroid 2-related factor 2 (Nrf2), a key regulator of oxidative stress. Nrf2 plays a crucial role in mitigating oxidative damage by activating antioxidant response elements and upregulating antioxidant enzymes such as superoxide dismutase (26). It has been demonstrated that Nrf2 is activated when TEAS is administered to patients undergoing surgery for gastrointestinal tumors. As a result, glutathione peroxidase 3 and superoxide dismutase could be expressed at higher levels. Additionally, TEAS could improve sleep quality, as evidenced by decreased Pittsburgh Sleep Quality Index and Athens Insomnia Scale scores. The mechanism may be related to the activation of the Nrf2/ARE signaling pathway and a reduction in the stress response (26).

Endothelin (ET) has vasoconstrictive and blood pressure-raising effects and can serve as an indicator of the intensity of the stress response (30). The application of TEAS during surgical anesthesia in elderly orthopedic patients could stabilize ET levels, thereby reducing intraoperative stress responses (31).

## 4 Alleviation of immune suppression

The activation and differentiation of T lymphocytes are crucial for the human body’s defense against infections and tumors (32). Cancer patients are commonly immunocompromised after surgical treatment, characterized by elevated CD8+ levels and decreased CD3+ and CD4+ levels. In a randomized controlled clinical trial, TEAS has been shown to reduce plasma CD8+ levels and increase plasma CD3+, CD4+, and the CD4+/CD8+ ratio, thereby enhancing the human body’s immune function (10, 33). In addition, the imbalance between T helper cells (Th1, Th2, Th17) and regulatory T cells (Treg) is mainly associated with immune dysregulation (34). Th1 cells counteract tumors by releasing immune cytokines such as γ-interferon (35). However, Th2 cells primarily participate in antibody-mediated immune responses. They collectively uphold the equilibrium of the immune system. Furthermore, Th17 cells and Treg cells have been defined as two distinct CD4+ T cell subsets derived from Th1 and Th2 cells (34). The expression levels of Th17-associated cytokines in patients with lung cancer were decreased following thoracotomy, which may have promoted an imbalance in numbers of Th1, Th2, Th17 and Treg cells, and contributed to postoperative immunosuppression. The use of TEAS during the perioperative period has been shown to increase Th1 and Th17 cell levels in lung cancer patients, promote the expression of related transcription factors, and enhance γ-interferon expression. Moreover, TEAS could reduce Th2 cells and decrease the expression of related transcription factors, partially restoring the balance of cell proportions, which may contribute to attenuating postoperative immunosuppression in lung cancer patients (35).

## 5 Regulation of endothelial function

Alterations in the production and availability of endothelial-derived nitric oxide (NO), endothelin (ET), and von Willebrand factor (vWF) are referred to as endothelial dysfunction and are considered a critical initiating factor in coronary artery disease. Among these, ET-1 and NO are vasoactive substances that regulate vasoconstriction and vasodilation, respectively, whereas vWF serves as an auxiliary factor in hemostasis. After percutaneous coronary intervention (36), endothelial cells are damaged, leading to a decrease in NO secretion and an increase in the levels of ET-1 and vWF. The application of TEAS at a frequency of 4 Hz during the perioperative period has been shown to suppress ET-1 and vWF levels in percutaneous coronary intervention patients, increase serum NO levels, and effectively improve endothelial function, thereby reducing (36).

A large number of studies have indicated that endothelial dysfunction and inflammatory responses could interact to form a vicious cycle, further exacerbating vascular damage and disease progression (36). Specifically, the inflammatory mediators act on the vascular endothelial cells, resulting in rearrangement of the actin filaments in the cytoskeleton, which subsequently induces cell contraction and morphological alterations (37). What’s more, the contraction of endothelial cells leads to the widening of intercellular gaps, which further facilitates the leakage of inflammatory mediators (38). In addition, CRP, as a highly sensitive and specific inflammatory marker, not only interacts with endothelial cells but also accelerates the vascular inflammatory process, leading to coronary plaque rupture. In the same clinical trial, TEAS not only regulated endothelial dysfunction but also modulated the levels of the inflammatory factor CRP. This suggests that TEAS may exert its therapeutic effects by simultaneously improving endothelial function and inhibiting the inflammatory response (36).

Additionally, endothelin (ET), as a vasoconstrictive peptide, is involved in the pathological processes of central nervous system damage. Calcitonin gene-related peptide (CGRP) could dilate cerebral vessels or counteract the vasoconstrictive effects of ET. ET and CGRP jointly maintain the vasomotor function of the cerebral vessels. Generally, after craniotomy, ET levels could be elevated, leading to an imbalance between CGRP and ET levels. Accordingly, the use of TEAS has demonstrated a reduction in ET levels among craniotomy patients, regulation of the CGRP/ET balance, and enhancement of microcirculation in brain tissue, thus offering protective effects against perioperative brain injury during neurosurgery (39).

## 6 Inhibition of coagulation and fibrinolysis system activation

When the damaged endothelial cells lose the physiological regulation mechanism of coagulation, the dynamic balance between coagulation, anticoagulation and fibrinolysis will be destroyed. This leads to hypercoagulability, intravascular microthrombosis, and disseminated intravascular coagulation (40). In this process, endothelial damage activates coagulation factor XII, triggering the intrinsic coagulation pathway. Endotoxins or tissue injury release tissue factor, which binds to and activates coagulation factor VII, thereby activating the extrinsic coagulation pathway and promoting thrombus formation (41). Furthermore, shortened prothrombin time, activated partial thromboplastin time, and elevated levels of D-dimer, fibrinogen, and cell aggregation are associated with an increased risk of thrombosis. The application of TEAS in perioperative patients for the prevention of deep vein thrombosis in the lower limbs could prolong prothrombin time and activate partial thromboplastin time, increase platelet count, and reduce D-dimer, fibrinogen, and red blood cell aggregation indices. These effects help alleviate the hypercoagulable state and improve blood circulation in the lower limbs (42, 43).

## 7 Discussion and perspectives

**Figure 1:**
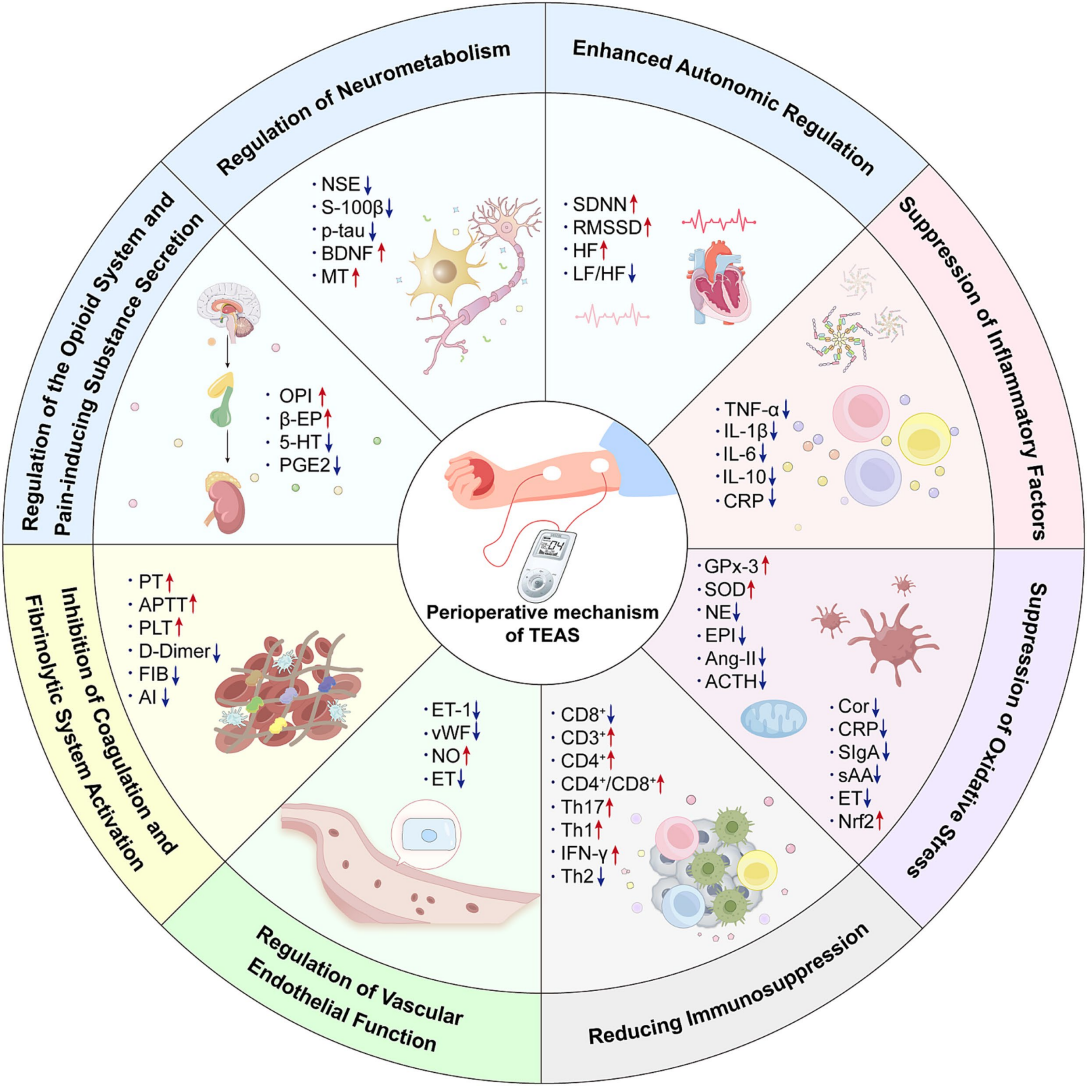
The mechanism of transcutaneous electrical acupoint stimulation in the perioperative period. OPI, Opiorphin protein; β-EP, β-Endorphin; 5-HT, 5-Hydroxytryptamine; PGE2, Prostaglandin E2; NSE, Neuron-specific enolase; S100β, Central nervous system specific protein β; p-tau, Phosphorylated Tau Protein; BDNF, Brain-derived neurotrophic factor; MT, Melatonin; SDNN, The standard deviation of the 24-h R-R interval; RMSSD, The root mean square of the adjacent R-R interval difference; HF, High-frequency power; LF, Low-frequency power; TNF-α, Tumor necrosis factor-α; IL-1, Interleukin-1; IL-6, Interleukin-6; IL-10, Interleukin-10; CRP, C-reactive protein; GPx-3, Glutathione peroxidase 3; SOD, Superoxide dismutase; NE, Norepinephrine; EPI, Epinephrine; AngII, Angiotensin-II; ACTH, Adrenocorticotropic hormone; Cor, Correlate; CRP, C-reactive protein; SIgA, Secretory immunoglobulin A; sAA, Salivary amylase; ET, Endothelin; Nrf2, Nuclearfactor erythroidderived 2-like 2; CD8+, Cluster of Differentiation 8 positive; CD3+, Cluster of Differentiation 3 positive; CD4+, Cluster of Differentiation 4 positive; Th17, Th17 cell; Th1, Th1 cell; IFN-γ, Interferon-γ; Th2, Th2 cell; ET-1, Endothelin-1; vWF, von Willebrand factor; NO, Nitric oxide; ET, Endothelin; PT - Prothrombin time; APTT, Activate partial thromboplastin time; PLT, Platelet count; FIB, Fibrinogen; AI, erythrocyte aggregation indices.

With extensive clinical research deepen, the therapeutic effects of TEAS during the perioperative period (3, 44, 45). To be specific, TEAS could be beneficial in several aspects, including anti-inflammatory, analgesic, stress-reducing, immune-enhancing effects, prevention of POCD, gastrointestinal regulation, and improvement of lower limb circulation (2). In addition, the mechanisms underlying these effects primarily focus on inhibiting inflammation, regulating the neuroendocrine system, modulating oxidative stress, alleviating immune suppression, improving endothelial function, and anticoagulation (A detailed summary is shown in Figure 1). These mechanisms play a crucial role in improving patient symptoms and enhancing overall prognosis during the perioperative period.

Although TEAS holds enormous promise for perioperative applications, its mechanism is still being developed. There still remains significant potential for advancement in mechanistic research. In particular, it could be manifested in the following aspects: (1) The mechanisms in the perioperative period typically involve the interconnection of inflammation, endothelial dysfunction, oxidative stress, and autonomic nervous system dysregulation. Studies have shown that increased sympathetic excitability may promote the occurrence of inflammation. However, current research on TEAS mechanisms tends to focus on a single aspect. Whether the suppression of perioperative inflammation by TEAS is closely related to autonomic nervous system regulation require further investigation. Clinically, trials of TEAS for perioperative patients may be designed to examine the impact of different autonomic nerve stimulation intensities on inflammation. Additionally, the pathological mechanisms of perioperative diseases are also associated with neuroinflammation, the release of harmful neurotransmitters, the activation mechanisms of microglia (46) and the expression of NLRP3 inflammasome (47). Specifically, microglia play a crucial role in neuroinflammation. In the state of neuroinflammation, they promote the release of inflammatory cytokines and trigger immune-inflammatory responses. NLRP3 inflammasome is one of the most typical inflammasomes involved in the inflammatory response, which may induce the activation of Caspase-1 and accelerate the maturation of pro-inflammatory cytokines such as IL-1β and IL-18. Nevertheless, whether TEAS could modulate these mechanisms remains to be explored in future studies. (2) The frequency of electrical stimulation in TEAS is a critical factor for its therapeutic effects, but there is still considerable debate regarding parameter selection. At present, commonly used frequencies in clinical studies include 2/100 Hz (15), 2 Hz (23), 100 Hz (48), and 4 Hz (36) (for more information on experimental design in literature, see supplementary documents). Some studies indicate that using a 2/100 Hz sparse-dense wave pattern during general anesthesia may better reduce stress responses in patients, increase postoperative motilin levels, and accelerate recovery (3). However, other studies reveal that 100 Hz TEAS is effective in alleviating postoperative nausea and vomiting symptoms in patients undergoing lung lobectomy (48). These parameters may have certain implications for mechanistic studies. In addition, variations in acupoint selection, stimulation parameters, sample sizes, and regional differences among research centers may also result in distinct physiological effects. Currently, there is a lack of standardized stimulation protocols in clinical practice. Given the inter-individual variability in parameter sensitivity, inconsistencies may exist in both the therapeutic efficacy of TEAS and its underlying mechanisms. Future research should aim to establish standardized parameters for TEAS in the perioperative period, so as to facilitate the clinical standardization of its application and clarify its mechanisms of action.

With the continuous integration and development of biomedical sciences, neuroscience, and electrophysiology, the application of TEAS in the perioperative period will become increasingly precise and efficient. Future research should focus on exploring the regulatory mechanisms of TEAS on neurotransmitter release during the perioperative period (49), the activation mechanisms of microglia, the expression of NLRP3 inflammasome, and the interactions between these mechanisms, among other aspects (50). Moreover, future studies should investigate the advantages of different perioperative conditions by employing various electrical stimulation frequencies of TEAS. By setting different electrical stimulation frequencies, the perioperative advantages of TEAS for various diseases could be explored. In addition, large-scale, multi-center clinical studies should be conducted to further standardize the clinical mechanism research of TEAS. These potential research directions will not only deepen our understanding of the mechanisms underlying TEAS but also provide safer, more effective, and convenient treatment options for perioperative patients, opening a new chapter of modern nerve electrical stimulation technology.
