# Supplementary material for: Research progress on the mechanism of transcutaneous electrical acupoint stimulation in the perioperative period
Source: Front Neurol. 2025 Apr 11;16:1563681. doi: 10.3389/fneur.2025.1563681 (PMC12021628; doi:10.3389/fneur.2025.1563681)
Supplement: Supplementary file 1 [file Table_1.docx]

Supplementary Material

# Supplementary Table

| Year | The type of trial | Duration of stimulation and total duration of treatment | Type of surgery | Electrode position | Number of people (control vs. intervention) | Intervention methods  (control vs. intervention) | Stimulation parameters | Ref. |
| --- | --- | --- | --- | --- | --- | --- | --- | --- |
| 2017 | Randomized Single-blind Controlled | 10 minutes before anaesthesia until end of surgery | Anorectal surgery | Neiguan (PC6)  Shenmen (HT7)  Shangliao (BL31)  Ciliao (BL32) | 146  （48/50/48） | Conventional drug anesthesia/  Conventional drug anesthesia and TEAS multiple acupoints/  Conventional drug anesthesia and TEAS two acupoints | Dense-sparse Waves, 2/100 Hz | [4] |
| 2021 | Randomized Controlled | 30 minutes before anaesthesia until end of surgery | Thoracoscopic lobectomy | Hegu (LI4)  Neiguan (PC6)  Zhigou (SJ6)  Chize (LU5) | 72  （36/36） | Conventional drug anesthesia/  Conventional drug anesthesia and TEAS | Dense-sparse Waves, 2/100 Hz,  The intensity of stimulation was twice the sensory threshold of the patient's acupuncture points | [5] |
| 2019 | Randomized Controlled | 30 minutes before anaesthesia until end of surgery | Thoracoscopic lobectomy | Hegu (LI4)  Neiguan (PC6)  Houxi (SI3)  Zhigou (SJ6) | 60  （30/30） | Sham TEAS/ TEAS | Dense-sparse Waves, 2/100 Hz, 6-12 mA | [6] |
| 2021 | Randomized Controlled | After the opening of the uterus is 3 cm, until the end of labor | Caesarean sections | Hegu (LI4)  Neiguan (PC6)  Jiaji  (EX-B2)  Ciliao (BL32) | 229（78/75/76） | No analgesic measures/  Patient-Controlled Epidural Analgesia Treatment/  TEAS | 2/100 Hz,  15-50mA | [7] |
| 2021 | Randomized Controlled | After surgery,30 minutes,  6 times  (3 days) | Gastric cancer surgery | Hegu (LI4)  Neiguan (PC6)  Weishu (BL21)  Xiaochangshu (BL27)  Zusanli (ST36)  Shangjuxu  (ST37) | 81  （40/41） | Conventional Surgery Treatment/  Conventional Surgery Treatment and TEAS | 20/100Hz | [8] |
| 2024 | Randomized Controlled | 30 minutes, 14 times  (7 days) | Gastric cancer surgery | Zusanli (ST36)  Neiguan (PC6)  Hegu (LI4) | 80  （40/40） | Sham TEAS/ TEAS | Dense-sparse Waves, 2/100 Hz,  0.2-0.6 ms, 8-12 mA | [10] |
| 2024 | Randomized Controlled | 30 minutes before anaesthesia until end of surgery | Total knee arthroplasty | Sanyinjiao  (SP6)  Neiguan (PC6)  Baihui (DU20) | 158（79/79） | Conventional drug anesthesia/  Conventional drug anesthesia and TEAS | Dense-sparse Waves, 2-100 Hz, 8-12 mA | [12] |
| 2022 | Observational research | 30 minutes before anaesthesia until end of surgery | Single-port total thoracoscopic lobectomy | Taiyang  (EX-HN5)  Fengchi (GB20) | 109（53/56） | Conventional drug anesthesia/  Conventional drug anesthesia and TEAS | Dense-sparse Waves, 2/100 Hz, ≤5mA | [13] |
| 2020 | Randomized Controlled | 10 minutes before anaesthesia until end of surgery | Gynecology, urology, etc | Yingxiang (LI20)  Yintang (DU29) | 40  （20/20） | Conventional drug anesthesia/  Conventional drug anesthesia and TEAS | Dense-sparse Waves, 2 /100 Hz | [15] |
| 2022 | Randomized Controlled | 30 minutes, 3 times (3 days) | Kidney transplantation | Shenmen (HT7)  Sanyinjiao (SP6)  Neiguan (PC6) | 60  （30/30） | Conventional drug anesthesia/  Conventional drug anesthesia and TEAS | Dense-sparse Waves, 2/100 Hz, 12-15mA | [16] |
| 2024 | Randomized Controlled | 30 minutes before anaesthesia until end of surgery | Laparoscopic cholecystectomy | Zusanli (ST36)  Tiantu (RN22)  Danzhong  (RN17) Zhongwan  (RN12)  Taichong (LR3)  Neiguan (PC6) | 204（68/68/68） | Conventional drug anesthesia/  Conventional drug anesthesia and TEAS two acupoints/  Conventional drug anesthesia and TEAS multiple acupoints | Dense-sparse Waves, 2/100 Hz, 5-15 mA | [17] |
| 2016 | Randomized Controlled | 30 minutes before anaesthesia until end of surgery; After surgery, 30 minutes, 6 times (3 days) | Abdominal surgery | Hegu (LI4)  Neiguan (PC6)  Zusanli (ST36) Sanyinjiao (SP6) | 58  （29/29） | Sham TEAS/ TEAS | Dense-sparse Waves, 2/100 Hz, 0.2-0.6 ms, 6-8 mA, 12-18 mA | [20] |
| 2023 | Randomized Controlled | 30 minutes before anaesthesia | Radical resection for esophageal cancer | Zusanli (ST36)  Hegu (LI4)  Feishu (BL13) | 64  （32/32） | Conventional drug anesthesia/  Conventional drug anesthesia and TEAS | Dense-sparse Waves, 2/100 Hz, 6-10 mA | [21] |
| 2017 | Randomized  Double-blind Controlled | 30 minutes before anaesthesia until end of surgery, After surgery, 30 minutes, 3 times (3 days) | Bowel resection surgery | Neiguan (PC6)  Hegu (LI4)  Zusanli (ST36) | 60  （30/30） | Sham TEAS/ TEAS | / | [22] |
| 2018 | Randomized Double-blind Controlled | 30 minutes before anaesthesia until end of surgery | Radical resection for colorectal cancer | Zusanli (ST36)  Hegu (LI4)  Neiguan (PC6) | 60  （30/30） | Conventional drug anesthesia/  Conventional drug anesthesia and TEAS | Continuous wave,  2 Hz, ≤ 2 mA | [23] |
| 2019 | Randomized Controlled | 20 minutes before anaesthesia until end of surgery | / | Quchi (LI11)  Zusanli (ST36) | 60  （30/30） | Conventional drug anesthesia/  Conventional drug anesthesia and TEAS | / | [25] |
| 2023 | Randomized Double-blind Controlled | 3 times (3 days) | Gastrointestinal tumor surgery | Shenmen (HT7)  Neiguan (PC6)  Zusanli (ST36) | 83  （43/40） | Sham TEAS/ TEAS | Dense-sparse Waves,  2/10 Hz ,6-15 mA | [26] |
| 2019 | Randomized Controlled | 30 minutes before anaesthesia | Cholecystectomy under general anesthesia | Hegu (LI4) Neiguan (PC6) Zusanli (ST36) | 122（61/61） | Sham TEAS/ TEAS | 2/100 Hz, 100 Hz  15-25 mA | [28] |
| 2023 | Randomized Controlled | 15 minutes before anaesthesia until end of surgery | Cholecystectomy | Neiguan (PC6)  Hegu (LI4)  Zusanli (ST36) Neiting (EX-LE6) | 100（50/50） | Conventional drug anesthesia/  Conventional drug anesthesia and TEAS | Before surgery：  2-10Hz After surgery：  4-6Hz | [29] |
| 2020 | Randomized Controlled | 5 minutes before anaesthesia until end of surgery | Orthopedic surgery | Neiguan (PC6)  Hegu (LI4) | 92  （46/46） | Conventional drug anesthesia/  Conventional drug anesthesia and TEAS | 2/100 Hz | [31] |
| 2014 | Randomized Controlled | 30 minutes before anaesthesia until end of surgery, After surgery, 30 minutes, 3 times (3 days) | Radical resection for lung cancer | Hegu (LI4)  Neiguan (PC6)  Houxi (SI3)  Zhigou (SJ6) | 40  （20/20） | Conventional drug anesthesia/  Conventional drug anesthesia and TEAS | Dense-sparse Waves, 2/100Hz  4-12mA | [33] |
| 2015 | Randomized Controlled | 30 min, prior to incision, and at 20, 44, 68, 92 and 116 h following thoracotomy | Thoracotomy for lung cancer | Hegu (LI4)  Neiguan (PC6) Houxi (SI3)  Zhigou (SJ6) | 81（27/27/27） | Conventional drug anesthesia/  Conventional drug anesthesia and TEAS | 2/100 Hz, 4-12 mA | [35] |
| 2021 | Randomized Controlled | 30 minutes before anaesthesia until end of surgery | Percutaneous coronary intervention | Neiguan (PC6)  Ximen (PC4) | 94  （47/47） | Sham TEAS/ TEAS | 4/20 Hz | [36] |
| 2008 | Randomized Controlled | 30 minutes before anaesthesia until end of surgery | Brain tumour surgery | Hegu (LI4)  Quchi (LI11)  Zusanli (ST36)  Sanyinjiao (SP6) | 50  （25/25） | Conventional drug anesthesia/  Conventional drug anesthesia and TEAS | 2/100 Hz, 8-12mA | [39] |
| 2019 | Randomized Controlled | 30 minutes preoperatively to 30 minutes postoperatively; After surgery, 30 minutes, 14 times (7 days) | Rectal cancer surgery | Diji (SP8)  Sanyinjiao (SP6)  Chengjin (BL56)  Zusanli (ST36) | 50  （25/25） | medical compression stockings/  medical compression stockings and TEAS | Dense-sparse Waves, 1-2 mA | [42] |
| 2017 | Randomized Controlled | 14 times (14 days) | Orthopedic surgery | Dubi (ST35)  Yinlingquan (SP9)  Yanglingquan (GB34)  Sanyinjiao (SP6)  Fenglong (ST40)  Weizhong (BL40)  Taichong (LR3) | 93  （47/46） | low molecular weight heparin calcium injection/  low molecular weight heparin calcium injection | / | [43] |
| 2023 | Randomized Controlled | 30 minutes before anaesthesia until end of surgery | lobectomy | Neiguan (PC6) | 66  （33/33） | Conventional drug anesthesia/  Conventional drug anesthesia and TEAS | Dense-sparse Waves, 2 Hz/100Hz, 5-15 mA | [44] |
